# Relationship between financial distress and mistreatment of workers regarding the COVID‐19 prevention measures: A 1‐year prospective cohort study

**DOI:** 10.1002/1348-9585.12403

**Published:** 2023-05-10

**Authors:** Naoya Sawamoto, Makoto Okawara, Ryutaro Matsugaki, Kiminori Odagami, Kosuke Mafune, Seiichiro Tateishi, Mayumi Tsuji, Akira Ogami, Yoshihisa Fujino, for the CORoNaWork Project

**Affiliations:** ^1^ Department of Environmental Epidemiology, Institute of Industrial Ecological Sciences University of Occupational and Environmental Health, Japan Kitakyushu Japan; ^2^ Department of Preventive Medicine and Community Health, School of Medicine University of Occupational and Environmental Health, Japan Kitakyushu Japan; ^3^ Department of Occupational Health Practice and Management, Institute of Industrial Ecological Sciences University of Occupational and Environmental Health, Japan Kitakyushu Japan; ^4^ Department of Mental Health, Institute of Industrial Ecological Sciences University of Occupational and Environmental Health, Japan Kitakyushu Japan; ^5^ Disaster Occupational Health Center, Institute of Industrial Ecological Sciences University of Occupational and Environmental Health, Japan Kitakyushu Japan; ^6^ Department of Environmental Health, School of Medicine University of Occupational and Environmental Health, Japan Kitakyushu Japan; ^7^ Department of Work Systems and Health, Institute of Industrial Ecological Sciences University of Occupational and Environmental Health, Japan Kitakyushu Japan

**Keywords:** COVID‐19, mistreatment, poverty, socioeconomic status (SES), workplace

## Abstract

**Objective:**

A substantial number of workers’ experience mistreatment in the workplace, impacting workers' health and companies' functioning. Vulnerability of those with lower income has been reported, yet little is known about mistreatment during COVID‐19. This study aims to examine whether workers in financial distress are particularly prone to mistreatment at the workplace with reference to pandemic‐related infection prevention measures.

**Methods:**

An internet‐based, year‐long prospective cohort study was conducted from 2020 to 2021. Participants were recruited from workers aged 20 and 65 years and currently employed at baseline. In total, 27 036 were included in the analysis and 18 170 responded to the follow‐up survey. The odds ratio (OR) of mistreatment at the workplace regarding COVID‐19 associated with the financial condition at baseline was estimated using multilevel logistic regression analysis nested by participant residence.

**Results:**

Compared with workers in a comfortable financial condition, those under financial stress showed significantly higher ORs of mistreatment (age‐ and sex‐adjusted model: 2.08, 95% confidence interval [CI] 1.75–2.47, *P* < .001, model adjusted for socioeconomic factors: 2.14, 95% CI 1.79–2.55, *P* < .001).

**Conclusion:**

Workers in financial distress were shown to be vulnerable to mistreatment at work regarding infection prevention measures in the COVID‐19 pandemic, underscoring a double burden of poverty and mistreatment. The perspective of vulnerable groups needs to be taken into account when implementing countermeasures against emerging infectious diseases, such as COVID‐19. As unfair treatment in the workplace might distort vulnerable employees' reactions to infection control (e.g., hiding infection), financial deprivation should be considered a public health issue.

## 1 INTRODUCTION

Mistreatment in the workplace, such as bullying (or mobbing) and incivility, is an important occupational health and safety issue because of its negative impact on workers' health and performance, as well as on the organization's functioning.^1^, ^2^ Bullying is a strongly deviant interpersonal behavior characterized by repeated and persistent negative acts.^3^ By comparison, incivility is defined as “low‐intensity deviant behavior with ambiguous intent to harm the target, in violation of workplace norms for mutual respect.”^4^ Although its intensity is less apparent than bullying, incivility also has a significant negative impact at work.^5^ Workplace mistreatment is associated with several negative consequences, such as psychological distress, increased tardiness and absenteeism, increased turnover and burnout, and decreased job satisfaction and commitment to the organization.^5^, ^6^, ^7^ A previous study showed that more than 70% of workers experience such mistreatment, making it a common and serious problem for workers.^8^

Currently, research on mistreatment focuses on its mechanisms and individual and organizational consequences.^3^ Numerous studies have shown a multidimensional and complex contextual framework that influences the emergence and persistence of mistreatment, including interactions among the main actors involved (perpetrator, target, and bystander), the work environment, and even social influences.^9^ The power imbalance between perpetrator and target has been shown to be an important factor.^10^ In fact, the definition of bullying usually does not include conflicts between individuals in an equal‐power relationship, but emphasizes the lower self‐defensive capability of the target.^3^ Power relationships involve not only positions such as manager and subordinate, but also gender, ethnicity, and employment status, as well as informal power relationships involving personal connections, skills, and possession of information.^11^

The power‐distance relationship is also found in stigma and prejudice against infectious diseases and other illnesses.^12^ Phelan and colleagues categorized stigma in terms of its function, whereby those with power attempt to preserve their position^13^: by stigmatizing those in weaker positions, those in power forcefully reinforce their relative positions. However, another function has been noted: the avoidance of disease, that is, recognizing disease as aversive and differentiating oneself from someone who is ill.^13^ Some authors have pointed out that underlying these acts is the fear of an unknown disease.^14^ Several studies have reported the stigmatization of vulnerable populations during outbreaks of “unknown” emerging infectious diseases, such as severe acute respiratory syndrome, N5H1, and COVID‐19.^12^

It has been suggested that the COVID‐19 pandemic in 2019 increased mistreatment related to infections in the workplace.^15^ In the early stages of the outbreak, amidst a flood of uncertain information about infectious diseases, negative social reactions, stigma, and prejudice against infected individuals and their families became widespread.^12^ Inappropriate interpersonal behaviors are likely to have occurred in the workplace as well, targeting employees and their families in relation to the infection. In Japan, the government recommended strong measures, such as curfews and behavioral restrictions, and required companies to take various infection control measures.^16^ However, the implementation of measures is highly dependent on the attitudes of individual companies, and some companies conceivably took excessive measures or reacted in ways that are perceived as unjustified.^16^

Based on previous studies, in the context of the COVID‐19 pandemic, it is predicted that socially vulnerable groups are prone to mistreatment at their workplace with respect to infection control measures.^17^ During the pandemic the number of financially distressed people increased, due to global economic stagnation.^18^ Workers with low socioeconomic status (SES) are known to be vulnerable to increased presenteeism, medical treatment interruptions, and other problems.^19^, ^20^ To date, however, few studies have examined the association with mistreatment related to infection control in the workplace.

Given that financial distress has been noted to reflect the reality of life more than SES,^21^ here, we tested the hypothesis that workers in financial distress are prone to experience mistreatment in the workplace regarding COVID‐19 prevention measures.

## 2 MATERIALS AND METHODS

An internet‐based prospective cohort study was conducted with follow‐up from December 2020 to December 2021. Participants were asked to complete online questionnaires at the start and end of the study. All participants received an explanation about the study's purpose and gave informed consent. The study was conducted as part of the Collaborative Online Research on the Novel‐coronavirus and Work (CORoNaWork) project and was approved by the Ethics Committee of the University of Occupational and Environmental Health, Japan (Ref. Nos. R2‐079 and R3‐006).

Details of the protocol are published elsewhere.^22^ The survey was commissioned by the company Cross Marketing Inc., which has 4.7 million pre‐registered monitors. Participants were workers aged between 20 and 65 years who were working when the study started (baseline). Stratified sampling was conducted taking into account geographic region, occupation, and sex. The regions were stratified into five levels according to the prevalence of COVID‐19 across the 47 prefectures of Japan. Occupations were categorized as office workers or non‐office workers. Thus, a total of 20 blocks were created combining 5 regions, 2 occupations, and 2 sexes. The number of participants in each block was planned to be approximately the same. To collect data from the originally planned total of 30 000 participants, approximately 600 000 people were sent a recruitment email. Of these, 55 045 were included in the initial screening, and 33 302 satisfied the final inclusion criteria. Of these 33 302 participants, 27 036 were included for analysis after excluding those judged as giving untrustworthy responses. These exclusion criteria are those employed in the Internet survey^23^ and included exceptionally short response times (≤6 min), reporting exceptionally low body weight (<30 kg) or exceptionally short height (<140 cm), and inconsistent responses to comparable questions (e.g., about marital status, or area of residence), as well as incorrect responses to a question designed to detect invalid responses (“Select the third largest number from among the following five numbers.”). At the 1‐year follow‐up survey, 18 170 participants were included after satisfying the inclusion criteria. There were no missing data, as this survey was internet‐based.

### 2.1 Financial condition

Financial condition was assessed at baseline using the same question as in the Japanese governmental survey^24^: How do you feel about your present livelihood situation financially? There were five response options: “very distressed,” “distressed,” “fair,” “somewhat comfortable,” “very comfortable.” To simplify the analysis, these five levels were then reduced to three as follows: if the response was “very distressed” or “distressed,” the participant was classified as “distressed;” “somewhat comfortable,” and “very comfortable” became “comfortable,” and “fair” was used as is.

### 2.2 Experience of workplace mistreatment regarding COVID‐19 prevention measures

Although there are various definitions of mistreatment, one widely accepted concept is the following: “a specific, antisocial variety of organizational deviance, involving a situation in which at least one organizational member takes counter normative negative actions—or terminates normative positive actions—against another member.”^25^ This study takes this perspective of “mistreatment” as a broad and general concept which can include various constructs, such as abuse supervision, aggression, bullying, harassment, interpersonal deviance, mobbing, ostracism, social undermining, and so on. As the definitions of such constructs also vary and there is some overlap among them,^26^ we do not focus on specific constructs in this study.

To our knowledge, there are no standard measures of mistreatment regarding infectious diseases, especially COVID‐19. In contrast, a plethora of measures has been used to study mistreatment.^27^ First, there is “self‐labeling”, which involves subjectively answering the simple question of whether or not one is experiencing mistreatment. Second, there is “describing behavioral items”, which involves asking behavior‐specific questions. Third, there is a composite of the two, employed in the present study. The questions were developed in consultation with occupational medicine specialists participating in the CORoNaWork project. For this study, we did not seek evidence of strong interpersonal deviant behavior; instead, the questions were designed to reveal mistreatment specifically regarding infection prevention measures, in line with recent research emphasizing the importance of how the target individual feels, that is, subjectivity.^26^ It has been shown that the target's sensitivity has a direct impact on their health.^3^ The present study, therefore, tried to capture the subjective aspect of mistreatment.

Experience of mistreatment at the workplace regarding COVID‐19 prevention measures was assessed by the following question: “Since January 2021, have you ever felt that you have been mistreated at your workplace for the following matters regarding COVID‐19, whether or not you were infected?” The question had the following 10 subquestions: “Q1. I had an unpleasant experience through conversation,” “Q2. The workplace asked me about my private friendships,” “Q3. The workplace asked me about my private behavior,” “Q4. The workplace asked me about my family,” “Q5. I was asked not to come to work for a certain period,” “Q6. I was ordered to work in another room,” “Q7. I was restricted from using common facilities,” “Q8. I was ordered to change my job duties and responsibilities,” “Q9. I was transferred to another workplace or reassigned to another department,” and “Q10. I was laid off.” All 10 subquestions were answered “Yes” or “No.” One or more “Yes” answers were taken to indicate the experience of mistreatment in relation to COVID‐19 prevention measures. Henceforth, “experience of mistreatment at the workplace regarding COVID‐19 prevention measures” is abbreviated as “workplace mistreatment regarding COVID‐19.”

### 2.3 Other covariates

The following participant characteristics were recorded at baseline as potential confounding factors related to financial distress and workplace mistreatment regarding COVID‐19^28^: age, sex marital status (married, divorced or bereaved, not married), job type (mainly desk work, mainly interpersonal communication, mainly manual labor), educational attainment (junior high school, high school, vocational school/college, university/graduate school), and a number of employees in the workplace (1–29, 30–99, 100–999, ≥1000).

### 2.4 Statistical analysis

We used multilevel logistic regression analyses to evaluate the relationship between financial conditions and workplace mistreatment regarding COVID‐19. The age‐ and sex‐adjusted and multivariate‐adjusted odds ratios (ORs) of the financial condition associated with mistreatment were estimated. In the multivariate analysis, we adjusted for the following socio‐economic factors: age, sex, marital status, job type, educational attainment, and a number of employees in the workplace. All analyses were nested in the prefecture of residence to take into account regional variability in COVID‐19 infection. *P* values below .05 were considered statistically significant. All analyses were conducted using Stata (Stata Statistical Software: Release 16; StataCorp LLC).

## 3 RESULTS

Table 1 summarizes basic participant characteristics according to the financial condition at baseline. A total of 7312 (40.2%) participants reported financial distress, while 2230 (12.2%) reported being in a comfortable condition. Better financial condition was associated with a higher level of education. Concerning job type, as financial conditions improved, the proportion of workers engaged in mainly desk work increased and the proportion of workers engaged in mainly labor decreased. More workers in better financial condition were employed by large companies (1000 or more employees) than by small companies (fewer than 100 employees).

**TABLE 1:** Basic characteristics according to financial condition at baseline.

	Financial condition		
	Distressed	Fair	Comfortable
	(*n* = 7312)	(*n* = 8628)	(*n* = 2230)
		*n* (%)	
Age, mean (SD)	48.8 (9.4)	48.2 (10.1)	48.7 (10.3)
Sex, male	4397 (60.1%)	4764 (55.2%)	1233 (55.3%)
Marital status, married	3860 (52.8%)	4969 (57.6%)	1415 (63.5%)
Job type			
Mainly desk work	3329 (45.5%)	4719 (54.7%)	1292 (57.9%)
Jobs mainly involving interpersonal communication	1873 (25.6%)	1993 (23.1%)	584 (26.2%)
Mainly labor	2110 (28.9%)	1916 (22.2%)	354 (15.9%)
Education			
Junior high school	145 (2.0%)	77 (0.9%)	15 (0.7%)
High school	2301 (31.5%)	2040 (23.6%)	345 (15.5%)
Vocational school/college, university, graduate school	4866 (66.5%)	6511 (75.5%)	1870 (83.9%)
Number of employees in the workplace			
1–29	2930 (40.1%)	2662 (30.9%)	613 (27.5%)
30–99	1086 (14.9%)	1368 (15.9%)	285 (12.8%)
100–999	1795 (24.5%)	2265 (26.3%)	581 (26.1%)
1000‐	1501 (20.5%)	2333 (27.0%)	751 (33.7%)

Table 2 shows frequencies of workplace mistreatment regarding COVID‐19 for workers according to their financial status at baseline. Overall, 10.9% of participants reported mistreatment, with those in financial distress reporting more than those who were financially comfortable (13.8% vs. 7.2%). This tendency was observed for all 10 questions covering mistreatment as defined in this study.

**TABLE 2:** Frequency of workplace mistreatment regarding COVID‐19 by financial condition at baseline.

			Financial distress			
			Distressed	Fair	Comfortable	Total
			(*n* = 7312)	(*n* = 8628)	(*n* = 2230)	(*n* = 18 170)
Workplace mistreatment regarding COVID‐19 (%)			13.8	9.4	7.2	10.9
Q1	I had an unpleasant experience in a conversation (%)		8.7	5.5	4.0	6.6
Q2	Workplace asked me about my private friendships (%)		2.7	2.1	1.3	2.3
Q3	Workplace asked me about my private behavior (%)		3.1	2.3	1.4	2.5
Q4	Workplace asked me about my family (%)		3.4	2.6	1.9	2.8
Q5	I was asked not to come to work for a certain period (%)		1.9	1.1	0.9	1.4
Q6	I was ordered to work in another room (%)		2.3	1.5	1.2	1.8
Q7	I was restricted from using common facilities (%)		1.1	0.7	0.5	0.8
Q8	I was ordered to change my job duties and responsibilities (%)		1.7	0.7	0.9	1.1
Q9	I was transferred to another workplace or reassigned to another department (%)		2.5	1.4	1.4	1.9
Q10	I was laid off (%)		1.5	1.1	1.0	1.2

Table 3 shows the ORs of workplace mistreatment regarding COVID‐19 by financial condition. Compared to those in a “fair” or “comfortable” financial condition, those who were “distressed” were significantly more likely to have experienced workplace mistreatment: OR in the age‐ and sex‐adjusted model of the financially distressed group was 2.08 (95% confidence interval [CI]: 1.75–2.47, *P* < .001). In the model adjusted for socioeconomic factors, OR of the financially distressed group was 2.14 (95% CI: 1.79–2.55, *P* < .001).

**TABLE 3:** Odds ratio of workplace mistreatment regarding COVID‐19 by financial condition at baseline.

	Univariate				Multivariate^a^			
	OR	95% CI		*P*	OR	95% CI		*P*
Financial condition								
Distressed	2.08	1.75	2.47	<.001	2.14	1.79	2.55	<.001
Fair	1.34	1.13	1.60	.001	1.33	1.12	1.59	.002
Comfortable	Reference				Reference			
Age	0.98	0.97	0.98	<.001	0.98	0.97	0.99	<.001
Sex								
Male	Reference				Reference			
Female	1.20	1.10	1.32	<.001	1.05	0.94	1.17	.402
Marriage status								
Married	Reference				Reference			
Divorced or bereaved	1.14	0.97	1.33	.105	1.08	0.92	1.27	.346
Not married	1.23	1.11	1.35	<.001	1.03	0.93	1.15	.555
Job type								
Mainly desk work	Reference				Reference			
Jobs mainly involving interpersonal communication	1.09	0.97	1.22	.159	1.06	0.95	1.20	.297
Mainly labor	1.28	1.14	1.43	<.001	1.17	1.05	1.32	.007
Education								
Junior high school	1.25	0.85	1.83	.258	1.12	0.76	1.65	.576
High school	1.05	0.94	1.16	.393	1.00	0.89	1.11	.932
Vocational school/college, university, graduate school	Reference				Reference			
Number of employees in the workplace								
1–29	0.76	0.67	0.86	<.001	0.70	0.61	0.80	<.001
30–99	1.17	1.01	1.35	.040	1.06	0.92	1.23	.420
100–999	1.25	1.10	1.41	.001	1.15	1.01	1.31	.029
1000‐	Reference				Reference			

^a^ Adjusted for age, sex, marital status, job type, educational attainment, number of employees in the workplace.

Table 4 shows the ORs for each question concerning workplace mistreatment regarding COVID‐19 and participants' financial condition. In the age‐ and sex‐adjusted model, the association between financial distress and responding “YES” was significant for all subquestions except Q6 (“I was ordered to work another room.”) In multivariate analysis, those in financial distress were significantly likely to answer “Yes” to all questions. The highest OR (2.45, 95% CI: 1.64–3.66, *P* < .001) was observed for Q5 (“I was asked not to come to work for a certain period.”) and the lowest (1.71, 95% CI: 1.08–2.71, *P* < .001) for Q6 (“I was ordered to work in another room.”)

**TABLE 4:** Odds ratio of questions concerning workplace mistreatment regarding COVID‐19 by financial condition at baseline.

		Age‐sex adjusted				Multivariate^a^			
Outcome	Financial condition	OR	95% CI		*P*	OR	95% CI		*P*
Q1. I had an unpleasant experience through conversation at work	Distressed	2.30	1.84	2.89	<.001	2.31	1.84	2.90	<.001
	Fair	1.38	1.10	1.74	.006	1.38	1.09	1.74	.006
	Comfortable	Reference				Reference			
Q2. Workplace asked me about private friendships	Distressed	1.91	1.27	2.88	.002	2.08	1.37	3.15	.001
	Fair	1.22	0.80	1.85	.360	1.26	0.83	1.91	.285
	Comfortable	Reference				Reference			
Q3. Workplace asked me about my private behavior	Distressed	1.86	1.33	2.59	<.001	1.93	1.38	2.69	<.001
	Fair	1.32	0.94	1.84	.107	1.33	0.95	1.86	.098
	Comfortable	Reference				Reference			
Q4. Workplace asked me about my family	Distressed	2.27	1.56	3.32	<.001	2.40	1.63	3.52	<.001
	Fair	1.62	1.10	2.37	.014	1.65	1.12	2.42	.011
	Comfortable	Reference				Reference			
Q5. I was asked not to come to work for a certain period of time	Distressed	2.20	1.48	3.28	<.001	2.45	1.64	3.66	<.001
	Fair	1.68	1.12	2.51	.011	1.75	1.17	2.62	.006
	Comfortable	Reference				Reference			
Q6. I was ordered to work in another room	Distressed	1.44	0.91	2.26	.116	1.71	1.08	2.71	.022
	Fair	1.01	0.64	1.60	.970	1.08	0.68	1.72	.738
	Comfortable	Reference				Reference			
Q7. I was restricted from using common facilities	Distressed	2.04	1.29	3.24	.002	2.28	1.43	3.64	.001
	Fair	1.19	0.74	1.90	.483	1.24	0.77	2.00	.369
	Comfortable	Reference				Reference			
Q8. I was ordered to change my job duties and responsibilities	Distressed	1.86	1.27	2.73	.002	1.98	1.34	2.92	.001
	Fair	0.99	0.67	1.48	.976	1.01	0.68	1.51	.952
	Comfortable	Reference				Reference			
Q9. I was transferred to another workplace or reassigned to another department	Distressed	1.84	1.14	2.96	.012	2.02	1.25	3.23	.004
	Fair	0.79	0.48	1.32	.347	0.83	0.50	1.38	.469
	Comfortable	Reference				Reference			
Q10. I was laid off	Distressed	2.01	1.09	3.71	.025	2.01	1.08	3.72	.027
	Fair	1.21	0.65	2.26	.553	1.19	0.64	2.23	.584
	Comfortable	Reference				Reference			

^a^ Adjusted for age, sex, marital status, job type, educational attainment, and number of employees in the workplace.

## 4 DISCUSSION

This study showed that workers experiencing financial distress were more likely to report mistreatment at their workplace regarding COVID‐19 infection countermeasures. ORs for most of the 10 questions covering workplace mistreatment tended to increase as financial conditions declined.

Several reasons for the connection between financial distress and mistreatment in the workplace may be proposed. In view of the lack of studies about workplace mistreatment in the context of COVID‐19, we discuss the topic with reference to research on workplace bullying, incivility, and stigma and prejudice associated with infectious diseases.

First, workers experiencing financial distress might occupy a relatively low‐ranking position in the workplace, making them easy targets of mistreatment. In workplace bullying research, bullying is an expression of power distance between the perpetrator and the target.^12^ In other words, workers with less authority (and usually lower salaries) are more likely to be bullied than those with higher authority (and higher salaries), such as managers. A similar pattern is found concerning discrimination and prejudice against infectious diseases.^12^ Those in power tend to sustain power differences by stigmatizing less powerful colleagues with regard to infectious diseases and emphasizing their control over them, thereby enhancing their self‐esteem and sense of self‐preservation while relieving anxiety about their own safety.^22^ These findings can help explain why those in lower‐status positions at work are more likely to receive mistreatment regarding COVID‐19.

Second, we assume that people in financial distress have more health problems, again making them more likely targets for mistreatment. Workers with physical or mental illnesses tend to receive lower salaries because of reduced working hours or absence from work. For example, psychological distress was identified as a risk factor for economic difficulties during the COVID‐19 pandemic.^29^ Workers with health problems also tend to “stand out” from other employees by working in a different way, which previous research has shown to increase vulnerability to bullying.^23^ Another study reported that physical illnesses may increase proneness to bullying in the workplace.^30^ Thus, it is expected that those with health problems are more likely to experience mistreatment.

Third, people in financial distress may be psychologically compromised, more sensitive to mistreatment, and thus further exposed to psychological burdens. The frustration hypothesis has been proposed as a factor explaining workplace bullying.^24^, ^25^ Specifically, workers of lower social status (assumed to experience more financial distress) experience more frustration because of impositions by those of higher status. This means that some workers may suffer psychologically from mistreatment even if infection control measures are reasonable and the demands legitimate. Moreover, those with low social status were found to receive less social support—a psychological buffer factor—for example, from their peer group.^31^, ^32^ Therefore, financially disadvantaged workers likely experience a greater psychological burden as a consequence of infection control measures.

The likelihood of experiencing all 10 circumstances used as examples of mistreatment in this study was significantly higher in financially distressed workers, a finding with potentially important implications for future infection control measures in the workplace. We have divided these circumstances into four categories to discuss what is required for the implementation of fair and balanced measures against workplace infection prevention‐related mistreatment.

### 4.1 Unpleasant conversations

Verbal aggression is a typical example of bullying and incivility in the workplace, with known negative impacts regardless of intensity.^17^ The present results concerning verbal aggression are consistent with previous reports of mistreatment directed toward those with lower economic status.^26^ Good leadership by executives and managers, through vigilance and positively influencing the organization's climate, has been shown to reduce mistreatment.^33^ Multiple studies also report that providing people with accurate knowledge about diseases prevents stigma and prejudice, suggesting the need for upper management to provide appropriate education to employees during a pandemic.^34^

### 4.2 Inquiring about private details

Excessive questioning by managers about an employee's personal life constitutes a form of “abuse management,” and has been studied as workplace mistreatment.^35^ During the COVID‐19 pandemic, the Japanese government conducted an active investigation of the transmission routes (The Active Epidemiological Investigation).^36^ Conceivably, concerns about infection within the workplace have grown sufficiently for infection transmission routes to be likewise investigated. In other words, those in higher positions, such as managers, may engage in private questioning of those in lower positions. Before research showed that managers' attitudes toward mistreatment could be either inhibitory or facilitatory; the typical example of mistreatment involved managers themselves becoming aggressors.^30^ Education on harassment is gradually spreading in Japanese workplaces; we propose that education on infection‐specific mistreatment by managers during a pandemic should be added to the list of topics.^37^

### 4.3 Restricting the use of facilities or asking to work in a different room

Restricting access to facilities and services is one of the most common expressions of stigma and prejudice in the context of infectious diseases.^14^ The risk of this form of mistreatment increases when quarantine and isolation are implemented, notably during outbreaks of serious infectious diseases when the public requires immediate information.^14^ Public health experts recognize that some groups are at high risk for these measures, and the results of the present study support this view.^7^, ^31^ Telecommuting or working from home (WFH) became widespread in Japan during the COVID‐19 pandemic. Although WFH is sometimes associated with increased psychological burden, it has been shown to be a buffer against bullying.^7^, ^38^ WFH should therefore be carefully considered as an alternative to restricting the use of facilities in the workplace. In addition to considering possible alternative working modalities, companies, and organizations should make efforts to promote accurate knowledge about infection control measures and emphasize fair treatment.

### 4.4 Changes in position or duties, or layoffs

It has been reported that low‐SES groups are more vulnerable with respect to employment during the COVID‐19 pandemic.^14^ This is consistent with the present findings: the workplace situation for economically vulnerable individuals may become worse within their companies, such as assignment to different positions or duties, even if they do not lose their jobs. This issue needs greater recognition as a public health issue concerning social vulnerabilities during a pandemic.

The perspectives of vulnerable groups should be taken into account the during implementation of countermeasures against emerging infectious diseases such as COVID‐19. Such countermeasures impose restrictions on people's lives for public health considerations, yet the impact of these measures is not uniform.^32^ The COVID‐19 pandemic revealed several issues. First, voluntary restraint on going out put considerable strain on some industries, including those involved in accommodation, and food production and sales (e.g., restaurants), especially for front‐line workers.^39^ Second, under the recommendation for telecommuting, the perceived vulnerability of specific occupations (e.g., the non‐feasibility of teleworking for manual labor workers) was revealed.^40^ These workers are mostly on low incomes, and this study has highlighted that they feel subjected to unfair treatment. Financially distressed workers suffer from a double burden: poverty, and being in a socially vulnerable position that leaves them open to unfair treatment at their companies. Possible outcomes of such mistreatment include affected workers being less likely to make truthful declarations about infections, and being forced to work under unhealthy conditions.^12^ They may also hesitate to access medical care, thereby potentially harming not only their personal health but also general public infection control measures.^17^ Financial deprivation needs to be considered as a public health issue that could have negative effects on society through how such workers are treated in their company.

Several limitations of this study are mentioned here. First, we used 10 limited‐content questions to investigate mistreatment regarding infection control. These were developed by a consensus of industrial hygiene experts, but there may be other types of mistreatment that were not measured in the survey. However, to our knowledge, there is no existing measure of mistreatment specifically concerning infection control. Second, we did not investigate the extent of mistreatment, as the answer field of questionnaire was YES or NO. However, even a relatively mild type of mistreatment, such as incivility, was shown to have negative impacts. We believe that the strengths of the study, which focused on these negative impacts, are not significantly diminished by the inclusion of some “mild” cases of mistreatment. Third, the occurrence of mistreatment was self‐reported. It is possible that reasonable measures taken by a company were reported as mistreatment. The frustration hypothesis discussed above indicates that those with low social status are prone to mistreatment, but may also inappropriately report reasonable control measures as mistreatment.^17^ Nevertheless, no matter how reasonable the treatment may be, the person's perception of being treated improperly, and its associated psychological distress, should be given due consideration.

## 5 CONCLUSIONS

Workers with financial distress were shown to be vulnerable to perceived mistreatment at work regarding infection prevention measures in the COVID‐19 pandemic, underscoring a double burden of poverty and mistreatment. The perspective of vulnerable groups needs to be taken into account when implementing countermeasures against emerging infectious diseases, such as COVID‐19. As unfair treatment in the workplace might distort vulnerable employees' reactions to infection control (e.g. hiding infection), financial deprivation should be considered a public health issue.

## AUTHOR CONTRIBUTIONS

**Naoya Sawamoto**: Conceptualization, investigation, data curation, and writing—original draft preparation. **Makoto Okawara**, **Kiminori Odagami**, **Ryutaro Matsugaki**, and **Kosuke Mafune**: Investigation, data curation, and writing—review and editing. **Seiichiro Tateishi**, **Mayumi Tsuji**, and **Akira Ogami**: Investigation, data curation, writing—review and editing, and funding acquisition. **Yoshihisa Fujino**: Conceptualization, methodology, software, formal analysis, investigation, data curation, writing—original draft preparation, project administration, and funding acquisition.

## FUNDING INFORMATION

This work was supported and partly funded by research grants from the University of Occupational and Environmental Health, Japan; the Japanese Ministry of Health, Labour and Welfare (H30‐josei‐ippan‐002, H30‐roudou‐ippan‐007, 19JA1004, 20JA1006, 210301‐1, and 20HB1004); Anshin Zaidan; the Collabo‐Health Study Group; and Hitachi Systems, Ltd.; and scholarship donations from Chugai Pharmaceutical Co., Ltd. The funders were not involved in the study design; the collection, analysis, and interpretation of data; the writing of this paper; or the decision to submit the paper for publication.

## CONFLICT OF INTEREST STATEMENT

The authors declare no conflict of interests for this article.

## DISCLOSURE

This study was approved by the Ethics Committee of the University of Occupational and Environmental Health, Japan (reference nos. R2‐079 and R3‐006). All participants provided informed consent after completing a form on the survey website.

## Data Availability

Data are available from the authors on reasonable request.
